# Miniaturization of hiPSC-derived 3D neural cultures in stirred-tank bioreactors for parallelized preclinical assessment of rAAV

**DOI:** 10.3389/fbioe.2024.1379597

**Published:** 2024-04-26

**Authors:** Catarina M. Gomes, Maria João Sebastião, Gabriela Silva, Filipa Moura, Daniel Simão, Patrícia Gomes-Alves, Paula M. Alves, Catarina Brito

**Affiliations:** ^1^ iBET, Instituto de Biologia Experimental e Biológica, Oeiras, Portugal; ^2^ Instituto de Tecnologia Química e Biológica António Xavier, Universidade Nova de Lisboa, Oeiras, Portugal

**Keywords:** gene therapy, stem cell bioengineering, miniaturization, stirred-tank bioreactor, CNS modeling, human induced pluripotent stem cell (hiPSC), recombinant adeno-associated viruses (rAAVs), 3D (three dimensional) models

## Abstract

**Introduction:** Engineered 3D models employing human induced pluripotent stem cell (hiPSC) derivatives have the potential to recapitulate the cell diversity and structure found in the human central nervous system (CNS). Therefore, these complex cellular systems offer promising human models to address the safety and potency of advanced therapy medicinal products (ATMPs), such as gene therapies. Specifically, recombinant adeno-associated viruses (rAAVs) are currently considered highly attractive for CNS gene therapy due to their broad tropism, low toxicity, and moderate immunogenicity. To accelerate the clinical translation of rAAVs, in-depth preclinical evaluation of efficacy and safety in a human setting is primordial. The integration of hiPSC-derived CNS models in rAAV development will require, amongst other factors, robust, small-scale, high-throughput culture platforms that can feed the preclinical trials.

**Methods:** Herein, we pioneer the miniaturization and parallelization of a 200 mL stirred-tank bioreactor-based 3D brain cell culture derived from hiPSCs. We demonstrate the applicability of the automated miniaturized Ambr^®^ 15 Cell Culture system for the maintenance of hiPSC-derived neurospheroids (iNSpheroids), composed of neuronal and glial cells. Critical process parameters were optimized, namely, cell density and agitation mode.

**Results:** Under optimized conditions, stable iNSpheroid cultures were attained in the microbioreactors for at least 15 days, with high cell viability and astrocytic and neuronal phenotype maintenance. This culture setup allowed the parallelization of different rAAVs, in different multiplicity of infections (MOIs), to address rAAV-host interactions at a preclinical scale. The iNSpheroids were exposed to rAAV2- and rAAV9-eGFP in the microbioreactors. Transgene expression was detected 14 days post-transduction, revealing different astrocyte/neuron tropism of the two serotypes.

**Discussion:** We advocate that the iNSpheroid cultures in miniaturized bioreactors are reliable and reproducible screening tools for addressing rAAV transduction and tropism, compatible with preclinical demands.

## Introduction

Gene therapy holds great potential to treat inherited central nervous system (CNS) diseases, offering the prospect of transformative and disease-modulating treatment opportunities with potential long-lived therapeutic effects. Recombinant adeno-associated viruses (rAAV) have been the most explored viral vectors for *in vivo* gene therapy, given the good safety profile, high efficacy, and long-term transgene expression demonstrated in preclinical studies (Nathwani et al., 2011); rAAV also showed lower immunogenicity in comparison to other viral vectors (reviewed in (Wang et al., 2019)). Another interesting feature of these vectors is their broad tropism, a result of the different naturally existent serotypes (Hammond et al., 2017; Gonzalez-Cordero et al., 2018). Nevertheless, translating the results from preclinical trials to effective therapeutics for humans has been a hurdle for gene therapy development, given limitations such as off-target effects and adaptive immune responses (cellular and humoral). These issues were not predicted in the preclinical development stage employing animal models (reviewed in (Colella et al., 2018; Rapti and Grimm, 2021; Bashor et al., 2022)). The clinical progress is further affected by the inability to directly compare data from different preclinical and clinical trials, owing to the different rAAV serotypes and variants employed, preclinical models used (Asokan et al., 2012), and patient-to-patient variability (Westhaus et al., 2023).

Preclinical assessment is typically performed in *in vivo* models that can represent complex cellular microenvironments and systemic interactions but differ in spatial distribution and cell-cell and virus-cell interactions (Khalil et al., 2020). These models often fail to recapitulate human-specific features, such as permissiveness to the vector, distribution of viral ligands, different viral tropism, human immune response, and human CNS-specific genetics. Altogether, these limitations hamper the study of transgene efficacy and safety. Additionally, animal model-based research is low throughput, expensive, and poses ethical constraints (Hammond et al., 2017; Gonzalez-Cordero et al., 2018). Innovation in experimental modeling of the brain is therefore needed to surpass these pitfalls. In recent years, human induced pluripotent stem cell (hiPSC)-derived three-dimensional (3D) models supported the study of biological processes by generating complex structures of neural cells that recapitulate cell-cell communication and cell-ECM interaction (Lancaster and Knoblich, 2014; Simão et al., 2018; Zhang et al., 2023; Zheng et al., 2023). These models can recapitulate the 3D neural development and maturation to different extents and can be employed for mechanistic studies and drug screening (Li and Izpisua Belmonte, 2019). When considering host-pathogen interactions, 3D models provide a versatile platform by enabling the study of pathogen diffusion, infectivity, and replication dynamics, in a simplified, robust, and scalable way (Brito et al., 2012; Piersanti et al., 2015; Simão et al., 2016a). Additionally, organoids were employed to generate knowledge on several viral features, such as trafficking and immune response, depicting important pathways for mitigating the pathogenicity of viral infection (Xu et al., 2019; Krenn et al., 2021; Wu et al., 2023). These 3D models offer promising approaches to complement animal models in preclinical research, decreasing costs and enabling screening capacity (Renner et al., 2020). Still, their integration in preclinical development will require, amongst other factors, robust, small-scale, high-throughput culture platforms that can feed the preclinical trials (Rafiq and Hewitt, 2015; Bédard et al., 2020; Khalil et al., 2020).

High-throughput technologies can improve efficiency, maximize screening potential while minimizing development resources, and reduce animal models use, thus reducing overall costs and time to market (Szymański et al., 2012; Fu et al., 2022). Additionally, they enable the use of Quality by Design approaches (e.g., factorial design of experiments, DoE) which identify the optimal conditions for the design, development, and manufacturing of advanced therapy medicinal products (ATMPs), reducing variability, increasing efficiency, leading to improved product quality (Bhambure et al., 2011; Martins Fukuda et al., 2018). The Ambr^®^ 15 Cell Culture is a high-throughput microbioreactor platform, which allows to parallelize up to 48 single-use stirred-tank bioreactors (STB), for which pH and gassing can be individually controlled, with automated online monitoring and control for pH, dissolved oxygen (dO2) (Rafiq et al., 2017). This platform has gained increasingly attention in an industry context, given its ability to perform cell line screening and media/feed development; growing evidence demonstrates the translation of results obtained in the Ambr^®^ 15 Cell Culture to larger scale stirred-tank systems (Grimm et al., 2015; Velugula-Yellela et al., 2018; Gagliardi et al., 2019; Fink et al., 2021; Wohlenberg et al., 2022). So far, the applications of the Ambr^®^ 15 Cell Culture have focused mostly on single cell suspension culture, with scarce examples of anchorage-dependent cell culture, using micro- or macrocarriers (Rafiq et al., 2017; Jayson et al., 2022).

This work aimed to adapt a hiPSC-derived 3D neural culture to a miniaturized bioreactor system, leveraging the use of advanced human cell models in preclinical development of advanced therapeutics. We employed the 3D human neurospheroid (iNSpheroid) model previously developed by our team (Bayó-Puxan et al., 2018; Simão et al., 2018). This comprises the use of 200 mL STB operated under perfusion, for 3D differentiation of hiPSC-derived neural precursor cells (hiPSC-NPCs) into iNSpheroids. This strategy allows co-differentiation of neurons, astrocytes, and oligodendrocytes; cells within the iNSpheroids to produce and deposit brain-like extracellular matrix (ECM), fostering cell-cell and cell-ECM interactions that recapitulate brain cell architecture features (Simão et al., 2018) and pathological processes (Bayó-Puxan et al., 2018). For miniaturization, we devised two main objectives: i) Optimize the transfer of human iNSpheroids cultures to the Ambr^®^ 15 Cell Culture platform; and ii) use the downscale model in a proof-of-concept study of rAAV tropism in a human neural cell context, prompting the translation of the model to preclinical development applications.

## Material and methods

### Human induced pluripotent stem cells (hiPSC) culture

The human iPSC(IMR90)-4 clone four line was acquired from WiCell Research Institute. The hiPSC were expanded on Growth Factor Reduced Matrigel Matrix (BD Biosciences) in mTeSR1 medium (StemCell Technologies) under feeder-free culture conditions. Complete medium exchange was performed every day. Cells were maintained under humidified atmosphere with 5% CO_2_, at 37 °C.

### Generation of human neural progenitor cells (hNPCs) from hiPSC

The iPSC(IMR90)-4 clone four line was plated at 0.8 × 10^5^ cell/cm^2^ in mTeSR1 and allowed to adhere. After 24 h, the culture medium was changed to DualSMADi medium, composition based on a previously published protocol (Chambers et al., 2009): N2B27 medium supplemented with SB431542 at 1 mM and LDN193184 at 10 mM (both from STEMCELL™ Technologies). N2B27 medium was composed by 50% DMEM/F-12% and 50% Neurobasal, N2-supplement (1x), B27 supplement without vitamin A (0.5x), GlutaMAX (1x), Penicillin-Streptomycin (1%), MEM Non-Essential Amino Acids Solution (1%) and 2-mercaptoethanol (50 μM) (all from Gibco™), and Insulin (20 μg/mL, Sigma-Aldrich). A 100% medium exchange was performed daily, for 10 days.

On day 6 or day 10, single cells (hNPCs) were collected and plated on poly-L-ornithine-laminin (PLOL)-coated surfaces, and kept in NPC expansion medium (EM): DMEM/F12 media with Glutamax (Life Technologies) supplemented with 1% N2 supplement (Life Technologies), 0.1% B27 supplement (Life Technologies), 1.6 μg/mL glucose (Sigma-Aldrich), 20 μg/mL insulin (Sigma-Aldrich), 20 ng/mL recombinant human basic fibroblast growth factor (rhu-bFGF, Peprotech) and 20 ng/mL human recombinant epidermal growth factor (EGF, Sigma-Aldrich). PLOL coating was prepared by performing a 3-h incubation at 37°C with 0.16 mg/mL solution of poly-L-ornithine in PBS (with Ca_2_^+^ and Mg_2_^+^), followed by a washing step and a 3-h incubation at 37°C with 1 μg/mL solution of laminin in PBS (with Ca^2+^ and Mg^2+^). The medium was renewed every other day until a confluent hNPC culture was obtained. For further expansion and maintenance, hiPSC-derived hNPCs (hiPSC-NPCs) were split typically every 4–5 days at 90%–100% confluence. Cells were dislodged by 0.05% Trypsin-EDTA, for 1–2 min, resuspended in DMEM supplemented with 10% fetal bovine serum (FBS, Life Technologies), sedimented by centrifugation and resuspended in NPC expansion medium. Cell concentration and viability were determined by the trypan blue exclusion method in a Fuchs-Rusenthal hemocytometer. hNPCs were plated in PLOL-coated T-flasks, at 3 × 10^4^ cell/cm^2^. A 50% media exchange was performed on day 2 of culture. Cells were maintained under humidified atmosphere, in a multi-gas cell incubator (Sanyo), with 5% CO_2_ and 3% O_2_, at 37°C.

### 3D differentiation of hNPCs derived from hiPSC (hiPSC-NPCs)

hiPSC-NPC differentiation in 3D was performed as previously described by our team (Simão et al., 2018). Briefly, the hiPSC-NPCs were expanded and harvested as described in the previous section. Before bioreactor inoculation, the cell suspension was filtered in a 70 µm nylon strainer (Millipore) to eliminate cell clumps and diluted to 4 × 10^5^ cell/mL in aggregation medium (AM). AM had the same composition as EM, with reduced concentration of EGF and bFGF (5 ng/mL) and 5 μM Y-27632. The single cell suspension was inoculated into a software-controlled stirred-tank DASGIP^®^ Bioblock bioreactor system (Eppendorf), from here onwards referred to as STB control. Culture conditions were set to maintain cells under 3% dissolved oxygen (15% of air with 21% of oxygen), pH 7.4, 37°C, and a stirring rate of 70–90 rpm. To control the aggregate size and avoid fusion, the stirring rate was gradually increased up to 90 rpm, with 10 rpm steps, based on visual inspection of the culture. After 72 h of culture, the perfusion operation mode was activated, with a dilution rate of 0.33 days^-1^ (i.e., 33% working volume exchange per day), under gravimetric control. To prevent the loss of aggregates through the outlet perfusion line, a metallic filter of 20 μm pore size was adopted as cell retention device. After a 7-day aggregation period in AM, differentiation was induced with differentiation medium (DM), for 23 days (a total of 30 days), to obtain iNSpheroids. DM: DMEM/F12 with Glutamax supplemented with 2% B27 supplement, 1.6 μg/mL glucose, 10 μg/mL insulin, 10 μg/mL putrescin, 63 ng/mL progesterone, 50 μg/mL apotransferrin, 50 ng/mL sodium selenium (all from Sigma-Aldrich) and 200 mM ascorbic acid (Wako).

### Ambr^®^ 15 Cell Culture Bioreactor system

Prior to inoculation, the vessels of the Ambr^®^ 15 Cell Culture Bioreactor (from here onwards referred to as Ambr15) were loaded onto the platform, and stabilized with respect to pH (7.4), temperature (37°C) and dO_2_ (15%). The working volume range was between 11 and 14 mL) and the impeller speed was set to 300 rpm, in down-pumping mode. The iNSpheroids were transferred from the 200 mL STB to the Ambr15 and cultured at three different cell densities: 0.5, 1.0 and 3.0 × 10^6^ cell/mL. Culture medium was composed of DMEM/F-12 without phenol red with 0.1X Glutamax, 2% B27 supplement, 1.6 μg/mL glucose, 10 μg/mL insulin, 10 μg/mL putrescin, 63 ng/mL progesterone, 50 μg/mL apotransferrin, 50 ng/mL sodium selenium (all from Sigma-Aldrich) and 200 mM ascorbic acid (Wako).

### Cell concentration and viability

Cell concentration and viability were measured using the fluorescence-based NucleoCounter^®^ NC-200™. For 3D culture characterization, reagent A100 and B were used to lyse the cells before nuclei concentration assessment using the ‘Viability and Cell Count—A100 and B Assay’ protocol.

Cell viability in intact iNSpheroids was assessed by incubation with 20 μg/mL fluorescein diacetate (FDA) in PBS, for viable cell labelling, and 10 μg/mL propidium iodide (PI) in PBS, a membrane-impermeable DNA-dye that labels non-viable cells. Samples were observed using a fluorescence microscope (DMI6000B, Leica).

### Metabolite analysis

Concentrations of glucose (Glc), glutamine (Gln), lactate (Lac), ammonia (NH_3_) and lactate dehydrogenase (LDH) in cell culture supernatants were measured using the Cedex Bio Analyzer (Roche). The metabolite consumption/secretion rate (qMet, expressed in pmol cell^-1^h^-1^) was calculated using the following equation:

$$q_{Met}=\frac{c_{Met}}{X_{v}∙\Delta_{t}}$$

where c_Met_ represents the variation on metabolite concentration with a specific cell concentration average, X_v_, during a time interval, Δ_t_.

### Immunofluorescence microscopy

iNSpheroids were harvested from the Ambr15 vessels and fixed in 4% paraformaldehyde (PFA) + 4% sucrose in PBS, for 20 min, at room temperature (RT), and washed three times with PBS. For immunostaining, blocking and permeabilization were performed simultaneously with 0.125% fish skin gelatine (FSG) and 0.1% or 0.5% Triton X-100 in DPBS (Supplementary Figures S1B, S1C, respectively), for 20 min at RT. Primary antibodies were then incubated overnight at 4°C diluted in 0.125% FSG +0.1% TritonX-100 in PBS. iNSpheroids were washed three times with PBS and incubated for 1 h with secondary antibodies diluted in the same solution as the primary. Primary antibodies were used as follows: anti-nestin (1:200, AB5922, Millipore), anti-βIII-tubulin (1:200, MAB1637, Millipore), anti-synaptophysin (1:200, MAB5258, Millipore), anti-MAP2 (1:200, Abcam), anti-MAP2 (1:200, ab92434, Abcam). The secondary antibodies were Alexa Fluor 488 goat anti-mouse IgG, Alexa Fluor 555 donkey anti-rabbit IgG, Alexa Fluor 647 goat anti-chicken IgY (1:500, A-11001, A-31572 and A-32933, Invitrogen). For labeling the cell nuclei, iNSpheroids were incubated with DAPI diluted in DPBS, for 5 min (1:1000, Life Technologies), and washed three times in DPBS. Coverslips were mounted in ProLong™ Gold Antifade Mountant (Life Technologies). Preparations were acquired in a Zeiss LSM 880-point scanning confocal microscope controlled with the Zeiss Zen 2.3 (black edition) software. Image acquisition was performed along the depth of the iNSpheroids, acquiring at least 35 sequential optical slices for each, starting in one of the ends and including the equatorial region to assure representation of at least half of the total volume of each spheroid. The obtained images were processed using ImageJ software version 1.53c (Schindelin et al., 2012) and only linear manipulations were performed.

### Viral vector production and purification

rAAV2 particles were generated in HeLaS3-rAAV2 producer (clone H32D2) cell line, purified and stored as described before (Escandell et al., 2023). Samples were boiled at 56 °C for 10 min prior to transduction, to eliminate any possible Adv contaminants. rAAV9 particles were supplied by the Bioproduction Unit of iBET; the vector was produced by triple transfection in the human embryonic kidney (HEK)293T expression system and purified by affinity chromatography. Concentration and buffer exchange was performed against PBS containing CaCl_2_ and MgCl_2_ (14040091, Gibco™). rAAV stock titers were determined by the real-time quantitative PCR (qPCR) titration method. Additionally, rAAV transduction efficiency was determined in the HT1080 cell line (ATCC^®^ CCL121) in 2D cultures (Supplementary Figure S4A–C). The hAdV5 vector was a E1-deleted human adenovirus type 5, based on plasmid pGS66, containing viral genome sequences from nucleotide 1 to 440 and 3523 to 35935; hAdV5 was produced using HEK293 followed by CsCl purification, as described before (Ferreira et al., 2009; Silva et al., 2015; Simão et al., 2016b). The multiplicity of infection (MOI) was determined as the number of viral genomes per cell (VG/cell) for rAAVs, and number of infectious viral particles per cell (ip/cell) for the hAdv5 control.

### rAAV transduction and tropism assessment

For both rAAV serotypes, two different MOIs were used, 10^**3**^ and 10^**4**^ viral genomes (VG) per cell for rAAV2 and 10^**5**^ and 5 × 10^**5**^ VG/cell for rAAV9, both encoding enhanced green fluorescent protein (eGFP). iNSpheroids were maintained in culture up to 14 days post-transduction, without medium exchange.

### RT-qPCR

Total RNA was extracted with High Pure RNA Isolation Kit (Roche) or Rneasy Mini Kit (Qiagen), according to the manufacturer’s instructions. RNA was quantified in a NanoDrop 2000c (Thermo Scientific) and used for cDNA synthesis. Reverse transcription was performed using the High-Fidelity cDNA Synthesis Kit (Roche), with anchored-oligo (dT)18 primer (Roche); for samples with low cDNA abundance, the Sensiscript RT Kit (Qiagen) was employed, with a mix between anchored-oligo (dT)18 primer and random hexamers (Roche). The qPCRs were performed in triplicates using LightCycler 480 SYBR Green I Master Kit (Roche) with the primers listed in Table 1. The reactions were performed in a LightCycler 480 Instrument II 384-well block (Roche). Quantification cycle values (Cq’s) and melting curves were determined using the LightCycler 480 Software version 1.5 (Roche). All data were analyzed using the 2^−ΔΔCT^ method for relative gene expression analysis (Livak and Schmittgen, 2001). Changes in gene expression were normalized using the housekeeping gene *RPL22* (ribosomal protein L22), *GADPH* (Glyceraldehyde 3 Phosphate Dehydrogenase), and *HPRT1* (Hypoxanthine Phosphoribosyltransferase 1) as internal controls.

**TABLE 1:** Primer sequences used for RT-qPCR.

Target gene	Forward (5’ → 3′)	Reverse (5’ → 3′)
*RPL22*	CACGAAGGAGGAGTGACTGG	TGTGGCACACCACTGACATT
*GAPDH*	AGAACATCATCCCTGCCTCT	ACCCTGTTGCTGTAGCCAAA
*HPRT1*	CCTGGCGTCGTGATTAGTGAT	AGACGTTCAGTCCTGTCCATAA
*GLUL*	CGTAGCTATCCGGACAGAGC	CCCAACCCCTACCTTCTCTC
*AQP4*	AGCAGTCACAGCGGAATTTCT	TCTGTTCCACCCCAGTTGATG
*GFAP*	AGAGAGGTCAAGCCCAGGAG	GGTCACCCACAACCCCTACT
*VIM*	GGAAACTAATCTGGATTCACTC	CATCTCTAGTTTCAACCGTC
*SIOOB*	GGAGACGGCGAATGTGACTT	GAACTCGTGGCAGGCAGTAGTAA
*TUBB3*	GGGCCTITGGACATCTCTTC	CCTCCGTGTAGTGACCCTTG
*SLCIA3*	ACCAGAAAACTGGGGCTCAA	GCAGGTTCTTGGAGGATTGC
*51W*	TGGAACAGGCAGAATTTTCA	GGACAACCTTTGTGCCATTC
*GFP*	GAACCGCATCGAGCTGAA	TGCTTGTCGGCCATGATATAG
*GADI*	ACCAGAAAACTGGGGCTCAA	GCAGGTTCTTGGAGGATTGC

*RPL22,* Ribosomal Protein L22; *GAPDH,* Glyceraldehyde 3-phosphate dehydrogenase; *HPRTI,* hypoxanthine phosphoribosyltransferase 1; *GLUL,* Glutamate-Ammonia Ligase; *AQP4,* Aquaporin-4; *GFAP,* Glial fibrillary acidic protein; *VIM,* Vimentin; *S100B,* S100 calcium-binding protein B; *TUBB3,* Tubulin Beta 3 Class III, *SLC1A3,* Solute Carrier Family 1 Member 3; SYN, Synaptophysin; *GFP,* Green fluorescent protein; *GAD1,* glutamic acid decarboxylase 1.

### Statistical analysis

Statistical comparisons between two experimental conditions were performed using paired *t*-test, while for three experimental conditions two-way ANOVA test with Šídák’s multiple comparison test was used: *p < 0.05; **p < 0.01, ***p < 0.001. Statistical significance was considered for *p* < 0.05. GraphPad Prism v10.1.2 (GraphPad Software, San Diego, CA) was used for data analyses and graphics.

## Results

### Critical process parameter modulation leveraged the transition of iNSpheroid cultures from STB to Ambr15

In this study, we leveraged from the human iNSpheroid model previously established by our team (Bayó-Puxan et al., 2018; Simão et al., 2018). hiPSC-NPC are cultured as neurospheres in STB and co-differentiation is induced by perfusion of neurotrophic agents until day 30 of culture, leading to neuronal and glial cells organized in 3D (iNSpheroids, Supplementary Figure S1A). iNSpheroids recapitulate features of the brain microenvironment, such as deposition of a human brain-like extracellular matrix and neuron-glia interactions, namely, by establishing functional glutamine-glutamate-GABA shuttle (Simão et al., 2018).

**FIGURE 1:**
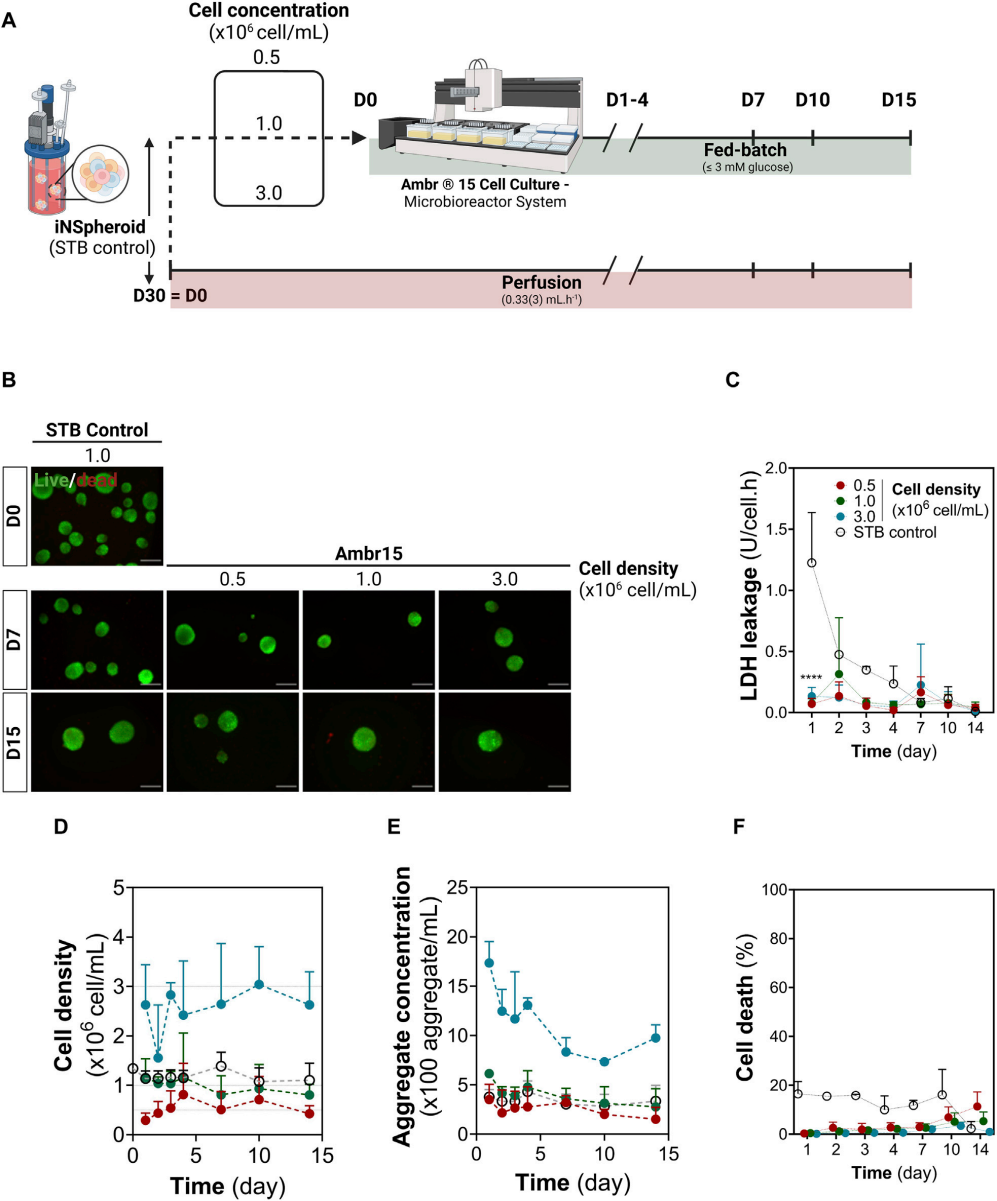
Establishing 3D human iNSpheroids cultures in the Ambr® 15 culture system (Ambr15). **(A)** Schematic representation of the experimental layout, comprising the transition of the iNSpheroid bioprocess to the Ambr15 system, from the 200 mL stirred-tank bioreactor (STB) to the Ambr15, with a parallel control culture in the STB. **(B)** Representative live/dead images of the iNSpheroids at the day of transfer from the STB to the Ambr15 (Day 0), and at 7 and 15 days after transfer. INSpheroids were stained with fluorescein diacetate (FDA, live cells, green) and propidium iodide (PI, dead cells, red). Scale bar, 100 μm. **(C)** LDH leakage assay, by measurement of LDH activity in culture supernatant; **(D)** Cell and **(E)** aggregate concentration throughout the different timepoints. Data are represented as mean ± SD of three independent experiments. **(F)** normalized cell death by assessing the ratio between LDH leakage in culture and total cell LDH, calculated by a calibration curve of total LDH in iNSpheroids.

After 30-day differentiation in 200 mL STB, iNSpheroids were transferred to the Ambr15 system or maintained in the STB (STB control). Five critical process parameters were considered given the differences and consequently scalability limitations between both systems: cell density, vessel hydrophobicity, impeller speed and direction, and perfusion. Three different cell densities were employed to test the versatility of the system: 0.5, 1.0, 3.0 × 10^6^ cell/mL (referred to as 0.5, 1.0, and 3.0 conditions), being 1.0 the cell density employed in the STB control (Figure 1A). Throughout the 14 days of culture, low to no loss of viability was observed by live/dead staining of cells in the iNSpheroids (Figure 1B), which correlated to a residual LDH leakage, indicating intact cell membranes (Figures 1C, D), similarly to what was observed the STB control. iNSpheroids are composed of fully differentiated neural cell lineages, which do not proliferate in homeostasis and therefore the cell concentration is maintained (Simão et al., 2018). Indeed, in conditions 0.5 and 1.0, cell and aggregate concentrations were constant throughout the 14 days of culture (Figures 1E, F), with no significant differences from the STB control. In condition 3.0, the standard deviations were higher for both variables, which led us to hypothesize that the hydrophobicity of the vessel surface, the mixing efficiency, the sampling volume and/or nutritional limitations could be critical factors.

After the 14 days of culture, vessels were emptied and incubated with crystal violet for detection of cell nuclei. In vessels from all conditions, cell deposition was restricted to the liquid-gas interface, with no crystal violet staining on the vessel walls or impellers (Supplementary Figure S1B), suggesting that the hydrophobicity of the vessel surface was sufficient to avoid deposition of iNSpheroids and single cells. As for mixing, a constant stirring of 300 rpm in down-pumping mode was employed; sampling volumes of 0.1, 0.2 and 0.45 mL were tested in all culture conditions, with coefficients of variation typically lower than 30% (Supplementary Figure S1C). Overall, these data indicated that the homogenization attained in the Ambr15 system is suitable for culture of iNSpheroids in the concentration range tested.

**FIGURE 2:**
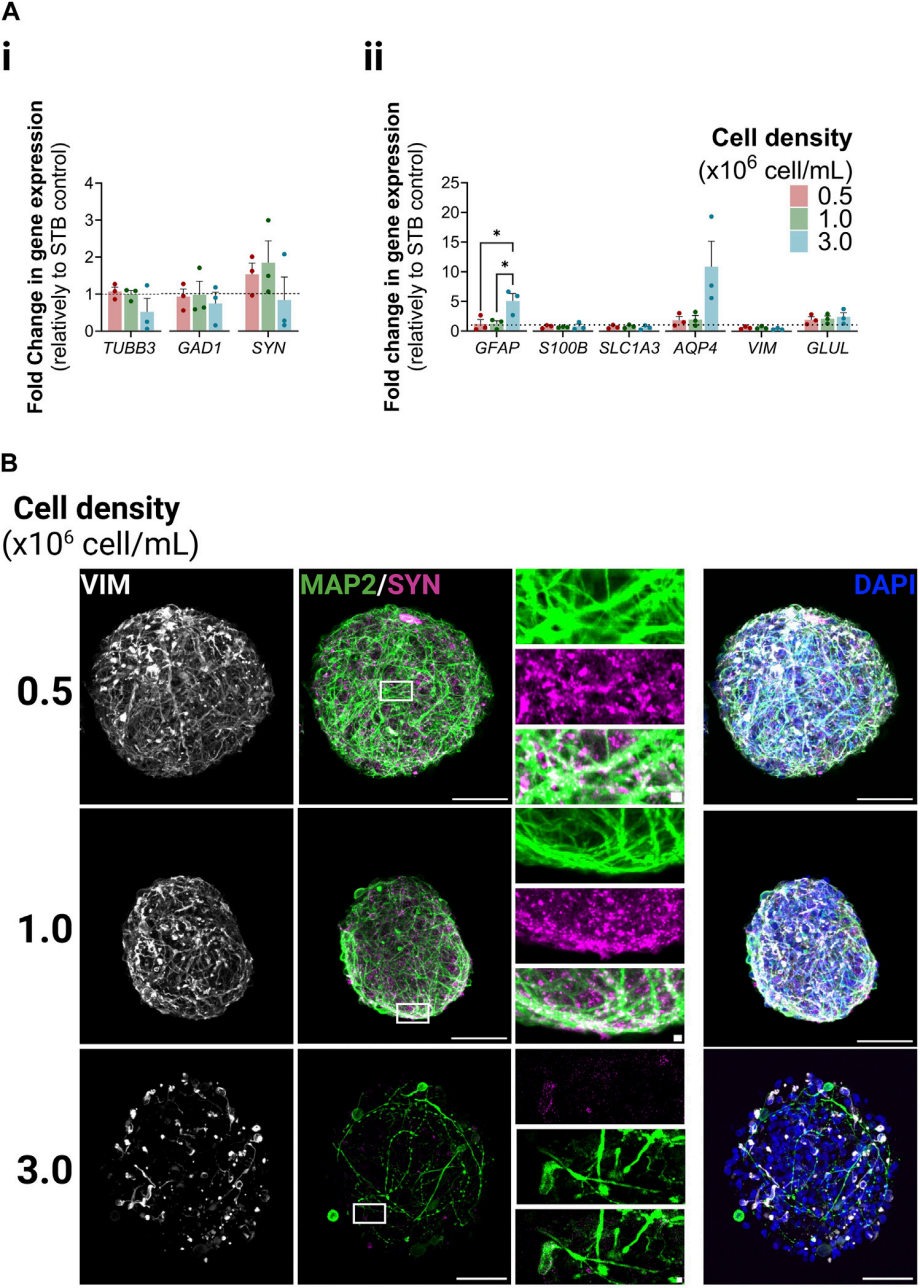
Neuron and astrocyte phenotype within iNSpheroids cultured in the Ambr^®^ 15 culture system (Ambr15). **(A)** Transcriptional profiles of neurons (i) and astrocytes (ii) after 15 days of culture in the Ambr15.; genes coding for neuronal β3-tubulin (*TUBB3*), glutamate decarboxylase (*GAD1*), and synaptophysin (*SYN*); and genes coding for astrocytic glial fibrillary acidic protein (*GFAP*), S100 calcium-binding protein B (*S100B*), glutamate-aspartate transporter 1 (*SLC1A3*), aquaporin-4 (*AQP4*), vimentin (*VIM*) and glutamine synthetase (*GLUL*) were analyzed by RT-qPCR and the comparative cycle threshold values method (2^−ΔΔCT^). Data are represented as mean ± SD of three independent bioreactor experiments. For comparing three or more groups two-way ANOVA test with Šídák’s multiple comparison test was used: *p < 0.05; **p < 0.01, ***p < 0.001. **(B)** Immunofluorescence detection of MAP2 (green, marker for somatodendritic compartments in mature neurons), (*SYN*) (magenta, marker for presynaptic vesicles of neurons) and vimentin (VIM) (white, marker for intermediate filaments in astrocytes) in the 0.5, 1.0 and 3.0 Ambr15 conditions introduce: right column correspond to the superimposition of the VIM, MAP2, SYN and DAPI signal. Scale bar, 50 μm; 2 μm for zoom-in insets.

We analyzed the expression of a panel of neuron- and astrocyte-specific genes. The expression levels of all genes analyzed were similar in iNSpheroids cultured in the STB control and in the 0.5 and 1.0 conditions; only for the 3.0 condition a tendency for downregulation of neuron-specific genes and an upregulation of astrocytic genes was observed. Glial fibrillary acidic protein (*GFAP*) and aquaporin-4 (*AQP4*), were, respectively, 5.09 ± 1.72 and 10.88 ± 6.06-fold higher in condition 3.0 than in the STB control (Figure 2A). Immuno-detection of microtubule-associated protein 2 (MAP2) and synaptophysin (SYN) in neurons revealed maintenance of typical cell morphology in the 0.5 and 1 conditions (Figure 2B, Supplementary Figure S2B). The juxtaposition of MAP2-positive dendrites and SYN-positive presynaptic terminals suggested that synapses were sustained in those conditions. Only in the 3.0 condition neuronal morphological changes were observed. There was a loss of complexity of the dendritic network, and low detection of SYN-positive presynaptic buttons. The latter did not accumulate near the somatodendritic compartment (identified by MAP2), suggesting neuronal functional loss in this condition. The astrocytic population was labeled with vimentin (VIM). Similar to neurons, the phenotype was maintained in the 0.5 and 1 conditions, while in the 3, dystrophic morphology and a decreased network complexity was observed. These features, together with *AQP4* and *GFAP* upregulation, suggest astrocyte activation (Liddelow et al., 2017; Neri et al., 2018; Guo et al., 2022).

Lastly, we evaluated the impact in iNSpheroids of transitioning from perfusion to fed-batch culture modes, since the Ambr15 is not coupled with a perfusion system; offline measurements of glucose (GLC), glutamine (GLN) and lactate (LAC) concentrations in the culture supernatant were performed along the 14 days. GLC feeds were done whenever the metabolite concentration was below 3 mM. No differences were found in consumption/production of these metabolites in the Ambr15 cultures, in comparison to the STB control, which indicated that the metabolic demands of the cells in iNSpheroids were fulfilled using fed-batch culture modes (Supplementary Figure S1D).

Altogether, these results demonstrate that the iNSpheroid cultures can be transferred to the miniaturized Ambr15 platform using a fed-batch approach. Moreover, all bioreactor cultures presented in this work and from follow-up studies ascend to nine independent transfers from STB control to Ambr15, using two different hiPSC lines (iPS(IMR90)-4 and iPS-DF6-9-9T.B), highlighting the robustness of the process (Supplementary Figure S2A).

### Parallel assessment of transduction and tropism of rAAV2 and rAAV9 in Ambr15 iNSpheroid cultures

**FIGURE 3:**
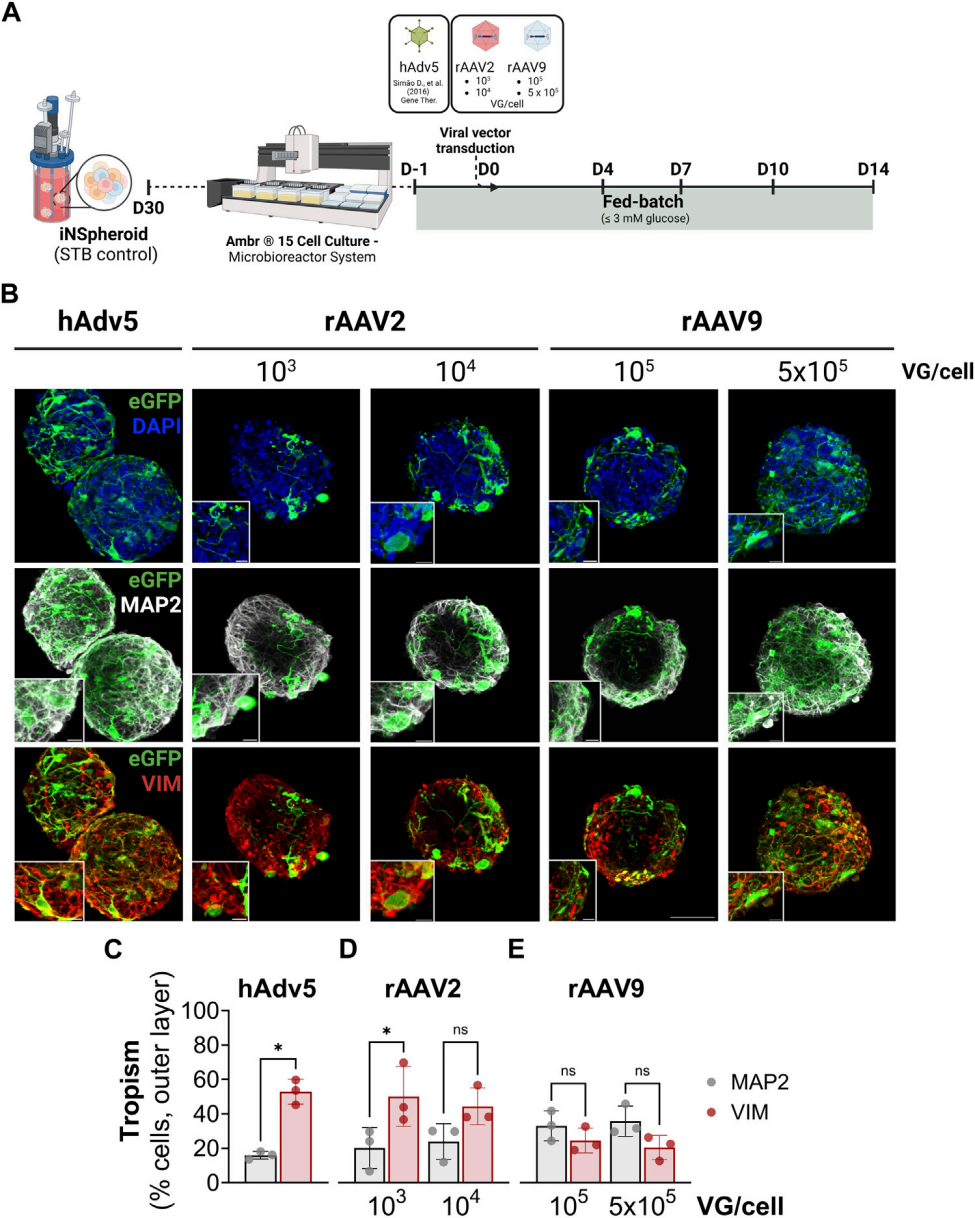
rAAV transduction of the iNSpheroids cultured in the Ambr^®^ 15 culture system (Ambr15). **(A)** Schematic representation of the experimental layout. INSpheroid were inoculated in the miniaturized system 1 day prior to the exposure to rAAV2-eGFP and rAAV9-eGFP and monitored for 14 days after transduction. **(B)** Immunofluorescence detection of eGFP (green), and the proteins microtubule-microtubule-associated protein 2 (MAP2, marker of the somatodendritic compartment of mature neurons, white), and vimentin (VIM, marker for intermediate filaments in astrocytes, red). Scale bar, 50 μm and 10 μm for zoom-in insets. Immunofluorescence-based quantification of eGFP, and MAP2 (neurons, white) or eGFP and VIM (astrocytes), using **(C)** hAdv5, **(D)** rAAV2 and **(E)** rAAV9 at the respective MOIs. Data are represented as mean ± SD, of three independent experiments. Statistical significance was evaluated by paired *t*-test: ns, non-significant; *p < 0.05.

To demonstrate that iNSpheroid cultures in Ambr15 are a suitable platform for preclinical assessment of advanced therapeutics targeting the human CNS, we addressed rAAV capsid tropism. We exposed the Ambr15 iNSpheroid cultures to rAAV2 and rAAV9 encoding eGFP under the control of the cytomegalovirus (CMV) promoter. Serotypes 2 and 9 have been the most used in clinical trials for CNS application, according to ClinicalTrials.gov (rAAV2: NCT04747431, NCT01621581, NCT02418598, NCT00229736, amongst others; rAAV9: NCT00876863 and NCT04127578). To characterize tropism, two MOIs were tested for both rAAV serotypes (rAAV2: 10^3^ and 10^4^ VG/cell; and rAAV9: 10^5^ and 5 × 10^5^ VG/cell, Figure 3A). The MOIs were selected based on literature, to attain comparable transduction efficiencies while avoiding vector-induced toxicity (Liu et al., 2022; Meumann et al., 2023). The hAdv5-GFP vector was used as a transduction control, at an MOI previously optimized (Simão et al., 2016b).

For all the viral vectors used, at all MOIs tested, there was detection of eGFP-positive cells at 14 days after viral exposure, indicative of transgene expression (Figures 3B–E). eGFP-positive cells were detected along the depth of the iNSpheroids (Supplementary Videos S1 and S2), more abundantly in the periphery than in the core of the 3D structures. Therefore, for tropism assessment, we performed image-based quantification of the transduced cells in the outer layer (40%) of the iNSpheroids. Tropism was evaluated by quantification of the percentage of MAP2-positive neurons also positive for eGFP, and VIM-positive astrocytes also eGFP-positive (Figures 3C–E and Supplementary Figures S3A, B). The rAAV2 employed transduced astrocytes more efficiently than neurons, with up to 50% of cells double positive for VIM and eGFP (50.1% ± 14.2% and 44.4% ± 8.7%, for MOIs of 10^3^ and 10^4^ VG/cell, respectively), *versus* less than 25% of cells double positive for MAP2 and eGFP (24.6% ± 5.9% and 20.5% ± 5.8%, respectively for rAAV2 10^3^ and 10^4^ VG/cell). As for the rAAV9, transduction efficiencies were not significantly different between the 2 cell types, although there was a tendency for higher tropism towards neurons (33.2 ± 7.1 and 35.8% ± 7.2% MAP2^+^eGFP^+^, for 10^5^ and 5 × 10^5^ VG/cell, respectively) than for astrocytes (19.8% ± 10.3% and 23.8% ± 8.7%, for 10^5^ and 5 × 10^5^ VG/cell, respectively). These differences in tropism were not a reflection of distinct transduction efficiencies, as there were no significant differences in the percentage of cells transduced by the two rAAV tested, at the two MOIs (Supplementary Figure S3B). The hAdv5, employed as control, transduced astrocytes with high efficiency and poorly transduced neurons (52.9% ± 5.9% VIM^+^eGFP^+^ cells and 16.0% ± 1.8% MAP2^+^eGFP^+^ cells (Figure 3C, Supplementary Figure S3B), in accordance with our previous data in human iNSpheroids derived from primary neural progenitor cells (Simão et al., 2016b).

**FIGURE 4:**
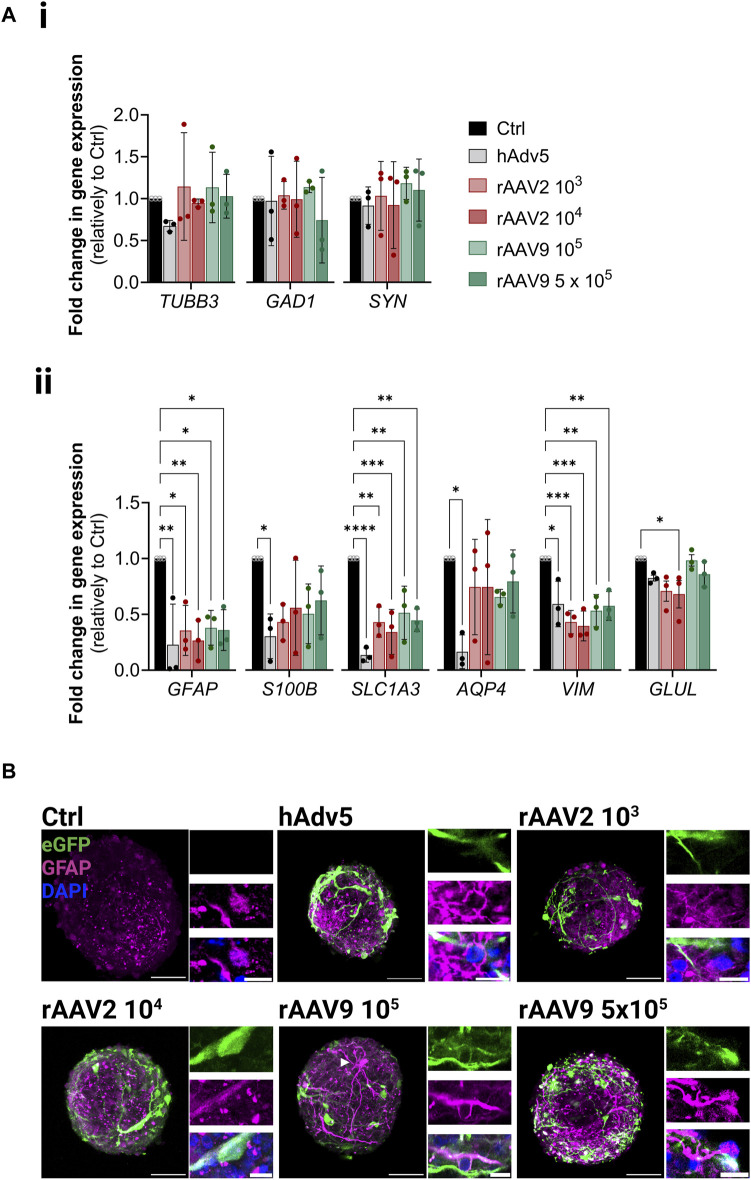
Neural cell phenotype modulation upon rAAV transduction in the Ambr^®^ 15 culture system (Ambr15). **(A)** Transcriptional profiles of (i) neurons and (ii) astrocytes, after 14 days of culture in the Ambr15; genes coding for neuronal β3-tubulin (*TUBB3*), glutamate decarboxylase (*GAD1*), and synaptophysin (SYN); and genes coding for astrocytic glial fibrillary acidic protein (*GFAP*), S100 calcium-binding protein B (*S100B*), glutamate-aspartate transporter 1 (*SLC1A3*), aquaporin-4 (*AQP4*), vimentin (*VIM*) and glutamine synthetase (*GLUL*) were analyzed by RT-qPCR and the comparative cycle threshold values method (2^−ΔΔCT^). Data are represented as mean ± SD of three independent bioreactor experiments. For comparing three or more groups two-way ANOVA test with Šídák’s multiple comparison test was used: *p < 0.05; **p < 0.01, ***p < 0.001. **(B)** Immunofluorescence detection of eGFP (green), GFAP (magenta) and DAPI (blue) in non-transduced control neurospheroids and neurospheroids transduced with the different viral vectors and MOIs. Scale bar, 50 μm; 2 μm for zoom-in insets.

To study the impact of rAAV transduction in the neural cells, we assessed the expression of the panel of neuronal and glial genes used in previous sections. Although no significant modulation of the neuronal genes analyzed was observed, there was a significant downregulation of glial genes upon transduction, namely, *GFAP* (3.5 ± 1.5 and 6.0 ± 4.3 reduction for rAAV2 10^3^ and 10^4^ VG/cell, respectively; 3.1 ± 1.3 and 3.2 ± 1.1 reduction for rAAV9 10^5^ and 5 × 10^5^ VG/cell, respectively) (Figure 4A). Nonetheless, for *S100β*, *GLAST* and *AQP4*, the modulations induced by both rAAV were significantly lower than those induced by hAdv5, for which astrocyte activation is well reported (Woo et al., 2017; An et al., 2021). Neglectable LDH leakage and propidium iodine incorporation in control and transduced iNSpheroids (Supplementary Figures S3C, D), together with no detectable differences between control and transduced iNSpheroids at the metabolic activity level (Supplementary Figures S3E, F), indicated that *GFAP* downregulation was not due to glial cell dead. Moreover, the presence of astrocytes with high intensity of GFAP fluorescent signal (GFAP^high^) and morphological alterations were common in iNSpheroids exposed to the viral vectors (Figure 4B, white arrowhead), whereas in control conditions GFAP^high^ astrocytes were not observed (Figure 4B). These data suggest that astrocytes may be activated by the rAAV, as it is described for adenoviruses, even that a lower extent. The iNSpheroid model in Ambr15 can leverage future studies to address astrocyte reactivity in response to rAAV.

## Discussion

In this study, we successfully transferred the human 3D iNSpheroids culture from a perfusion 200 mL STB to a microbioreactor system, leveraging these cultures as a screening tool for preclinical assessment of ATMPs. This strategy allows the large-scale production of iNSpheroids from a uniform batch, with minimal manipulation and disturbance of the environment over 30 days using a perfusion-operating mode. These feed high-throughput systems for parallelized ATMP screenings on a smaller scale, as demonstrated here for rAAV. This approach reduces the need for continuous and parallel quality control of differentiation, which makes the entire process more efficient and cost-effective.

We identify critical process parameters that could impair the transfer of the iNSpheroid model from the DASGIP^®^ Parallel Bioreactor Systems to the Ambr^®^ 15 Cell Culture, namely, the cell density and impeller speed. We demonstrated that, in optimized conditions we obtained a homogeneous distribution of the iNSpheroids, without loss of cell viability, cell or aggregate concentration, and no cell deposition or adhesion in the vessel. Both the 1.0 and 0.5 conditions maintained neuronal and glial phenotypes and morphologies as in the 200 mL STB, evaluated by both gene expression and protein detection. Notably, we could attest the robustness of the system, with nine out of nine successful transfer and culture of iNSpheroids (using two hiPSC lines, iPS(IMR90)-4 and iPS-DF6-9-9T.B, Supplementary Figure S2A) in Ambr15 for at least 10 days. Nonetheless, for applications that require higher cell densities, an in-depth study should be performed, given that our results suggest neuronal damage and astrocyte reactivity in the higher cell density condition (3.0). Legrand et al., described that working volume, impeller speed and direction, aeration and suspension density can greatly influence the flow profile inside the Ambr15 vessels (Rafiq et al., 2017; Legrand et al., 2022). Suspension density was described as having the greatest influence on the bulk liquid mixing and creation of dead-zones, which is critical when employing down-pumping. Although operating in down-pumping mode, no cell deposition or shear stress induced cell death was observed in our vessels, independently of the cell density. This modality has been often used for dense particle suspension including microcarriers culture (Ibrahim and Nienow, 2004; Rafiq et al., 2017), enabling their suspension with lower mean specific energy dissipation rate compared with up-pumping, as well as similar mixing times (Nienow, 1997; Rafiq et al., 2017). Nevertheless, scale-down of hiPSC-derivatives can be problematic owing to the complexity of the parameters involved not allowing for a generalized solution. We emphasize the need for in depth process understanding, with characterization of cell culture environment that goes beyond nutritional limitations and central carbon metabolism, as other soluble factors involved in cell communication, from differentiation and growth factors to cytokines and chemokines, play critical roles in the vitality of complex co-cultures.

In preclinical studies, rAAVs were shown to be powerful tools for gene delivery demonstrating long-term transgene expression and inducing negligible levels of immunogenicity in comparison to other viral-based gene therapy vectors. However, clinical studies have demonstrated significant limitations of these viral vectors. These limitations include distinct transduction efficiency and tropism in patients, when compared to the animal models; adaptative immune response, humoral and cellular (presence of neutralizing antibodies or T cell-mediated responses) (Manno et al., 2006; Mingozzi et al., 2007; Flotte et al., 2011; Markusic and Herzog, 2013; Hui et al., 2015). Given the fundamental differences between animal models and human patients, it is critical to demonstrate the efficacy and safety of such vectors in human-relevant models to boost their use in clinics. Here we provide a proof-of-concept for utilizing this miniaturized system for better understanding vector tropism, and cell toxicity of gene therapy rAAV. We analysed rAAV of serotypes 2 and 9, which have been investigated for CNS applications given their broad tropism (rAAV2) and ability to cross the blood-brain-barrier (rAAV9) (reviewed in (O’Carroll et al., 2021; Srivastava, 2016). Indeed, rAAV9 has been used in an FDA approved gene therapy, Zolgensma, to deliver a functional gene encoding the survival of motor neuron (SMN) protein to motor neuron cells in spinal muscular atrophy. Our results show that both viral vectors transduce the neural cells of iNSpheroids, however rAAV2 showed a higher tropism towards glia cells (up to 50% of astrocytes in culture expressing eGFP), while rAAV9 had similar tropism for neurons and astrocytes 25%–30% of MAP2- and eGFP-double positive cells). These results are in line to previous reports in animal studies (Gray et al., 2013; Liu et al., 2022; Markakis et al., 2010). No significant difference in transduction efficiency was observed between the two MOI tested, for either rAAV serotype. On the other hand, for each of the viral vectors, we observed differences in transduction efficiency by using a dynamic and static system (Supplementary Figure S4), as well as in the outer and inner layers of the iNSpheroids. The iNSpheroid data emphasize the need for a better understanding of the biological factors governing rAAV transduction, and the suitability of the iNSpheroid model for these preclinical studies. In a previous study, we explored iNSpheroids to distinguish not only tropism but also tissue penetration of a helper-dependent canine adenovirus *versus* human adenoviral vector (Simão et al., 2016a). No significant cell toxicity was observed using both rAAV serotypes, independently of the MOI. This result goes along with previous reports on rodent CNS cells, showing that rAAV2 and rAAV9 are the least toxic serotypes (Howard et al., 2008). However, when addressing the impact of transduction on neural cells, we observed a significant downregulation of astrocytic genes, namely, *GFAP*, *GLAST*, *VIM* and, to less extent, *GLUL*, for both rAAV serotypes and hAdv5. At the protein level, we could detect astrocytes with very high GFAP levels. Modulation of the expression of these genes have been linked to astrocyte response to inflammatory stimuli, namely, pathogen infection (Ding et al., 2021; Palacios et al., 2023; Rubio-Hernández et al., 2023). Moreover, it is well established that adenoviruses lead to astrocyte reactivity, characterized by increased GFAP (Woo et al., 2017; An et al., 2021). Most of the reports addressing astrocyte reactivity (which rely on cytokines to induce this state) showed an upregulation of *GFAP* and *VIM* at very early timepoints, while function-related genes (*GLAST*, glutamate receptor, and *GLUL*, glutamine synthase) were downregulated. Nonetheless, studies on infection of astrocytes by the ZIKA virus reported the downregulation of these genes, 5–6 days after infection (Howard et al., 2008; Rubio-Hernández et al., 2023), suggesting the reestablishment of homeostasis by modulation of the acute inflammatory response occurring after viral infection. The temporal dynamics of astrocytic gene modulation induced by the viral vectors may explain the detection of GFAP^high^ astrocytes 14 days after detection, whereas *GFAP* expression levels at that point were already downregulated. Further studies will be required to address astrocytic activation upon rAAV transduction.

hiPSC-derived 3D models represent a new avenue for personalized medicine and disease modelling as skin biopsies can be collected, differentiated, and matured into any cell of the central nervous system and tested for ATMP therapeutic efficacy and toxicity (reviewed in (Tang et al., 2022)). Besides better representing the human context, these models can be easily manipulated and maintained, are potentially scalable, and have a lower cost. Beyond these advantages, hiPSC technology enables to derive patient-specific organoids maintaining the genetic background. Our study is timely providing a robust and well characterized platform to address the current major efforts in the field, such as specificity of new promoters and rAAV capsid engineering developments. Our work opens a new avenue for assessing the human-specific efficiency and safety of therapeutic transgenes and formulations. In summary, we have demonstrated that the iNSpheroid culture can be transferred to a miniaturized system, broadening the application of this model as a screening platform to prompt ATMP development. This strategy can be extended to other cell sources, namely, patient-derived iPSC to give a more reliable perspective of the genetic background of the patient and predict gene therapy outcome.

## Data Availability

The original contributions presented in the study are included in the article/Supplementary Material, further inquiries can be directed to the corresponding author.
