# Supplementary material for: Miniaturization of hiPSC-derived 3D neural cultures in stirred-tank bioreactors for parallelized preclinical assessment of rAAV
Source: Front Bioeng Biotechnol. 2024 Apr 26;12:1379597. doi: 10.3389/fbioe.2024.1379597 (PMC11082387; doi:10.3389/fbioe.2024.1379597)
Supplement: Supplementary file 3 [file DataSheet1.docx]

Supplementary Material

Miniaturization of hiPSC-derived 3D neural cultures in stirred-tank bioreactors for parallelized preclinical assessment of rAAV

Catarina M. Gomes^1,2^, Maria João Sebastião^1^, Gabriela M. Silva^1^, Filipa Moura^1,2^, Daniel Simão^1^, Patrícia Gomes-Alves^1^, Paula M. Alves^1,2^, Catarina Brito^1,2,*^

^1^iBET, Instituto de Biologia Experimental e Biológica, Oeiras, Portugal

^2^Instituto de Tecnologia Química e Biológica António Xavier, Universidade Nova de Lisboa, Oeiras, Portugal

*** Correspondence:**Catarina Brito
anabrito@ibet.pt

# Supplementary Figures


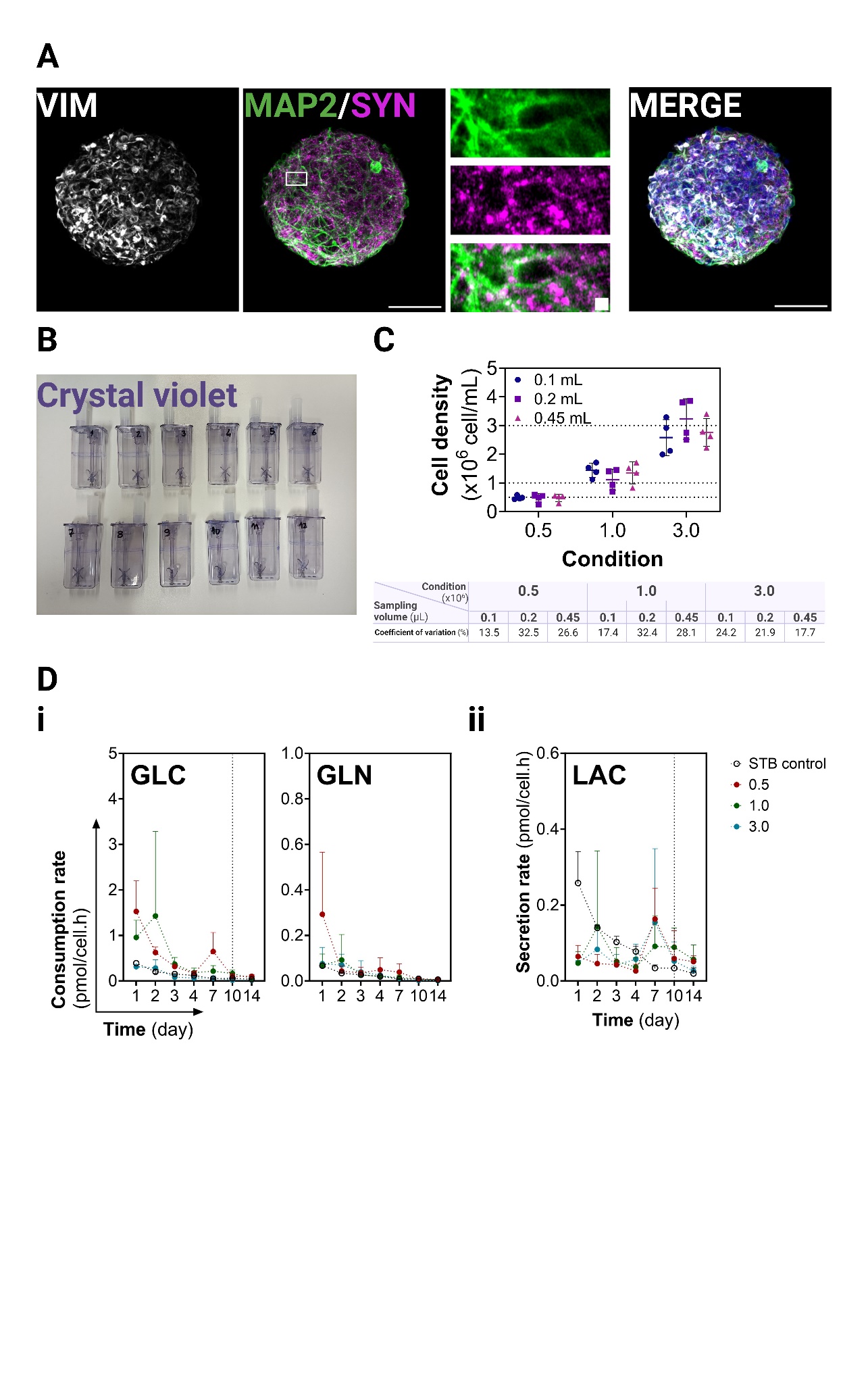


**Supplementary Figure 1 - Establishing 3D human iNSpheroids cultures in the Ambr® 15 culture system.**

**(A)** Immunofluorescence detection of MAP2 (green, microtubule-associated protein 2, marker of the somatodendritic compartment of mature neurons), synaptophysin (magenta, component of presynaptic vesicles of neurons) and vimentin (white, intermediate filament of astrocytes), in iNSpheroids generated in 200 ml stirred tank bioreactors (day 30). Maximum intensity projection of ten consecutive optical slices of 2 µm. Representative pictures depicting one of 3 independent experiments. Scale bar, 50 μm; 2 μm for zoom-in insets. **(B)** Ambr15 vessels stained with crystal violet (purple) to detect cellular DNA. **(C)** Cell concentration along culture time, employing different Ambr15 sampling volumes. Data from one independent experiment, with 3 sampling volumes (0.1, 0.2, 0.45 mL) for each bioreactor (0.5, 1 and 3 conditions), at 4 different sampling points (1-, 4-, 7- and 10-days post-inoculum). Mean, standard deviation (SD) and coefficient of variation determined for each sampling volume (N=4). **(D)** Specific **(i)** glucose (GLC) and glutamine (GLN) consumption; and **(ii)** lactate (LAC) secretion**.** The vertical dashed line represents the timepoint of the feeding. Data represented as mean ± SD of three independent experiments.


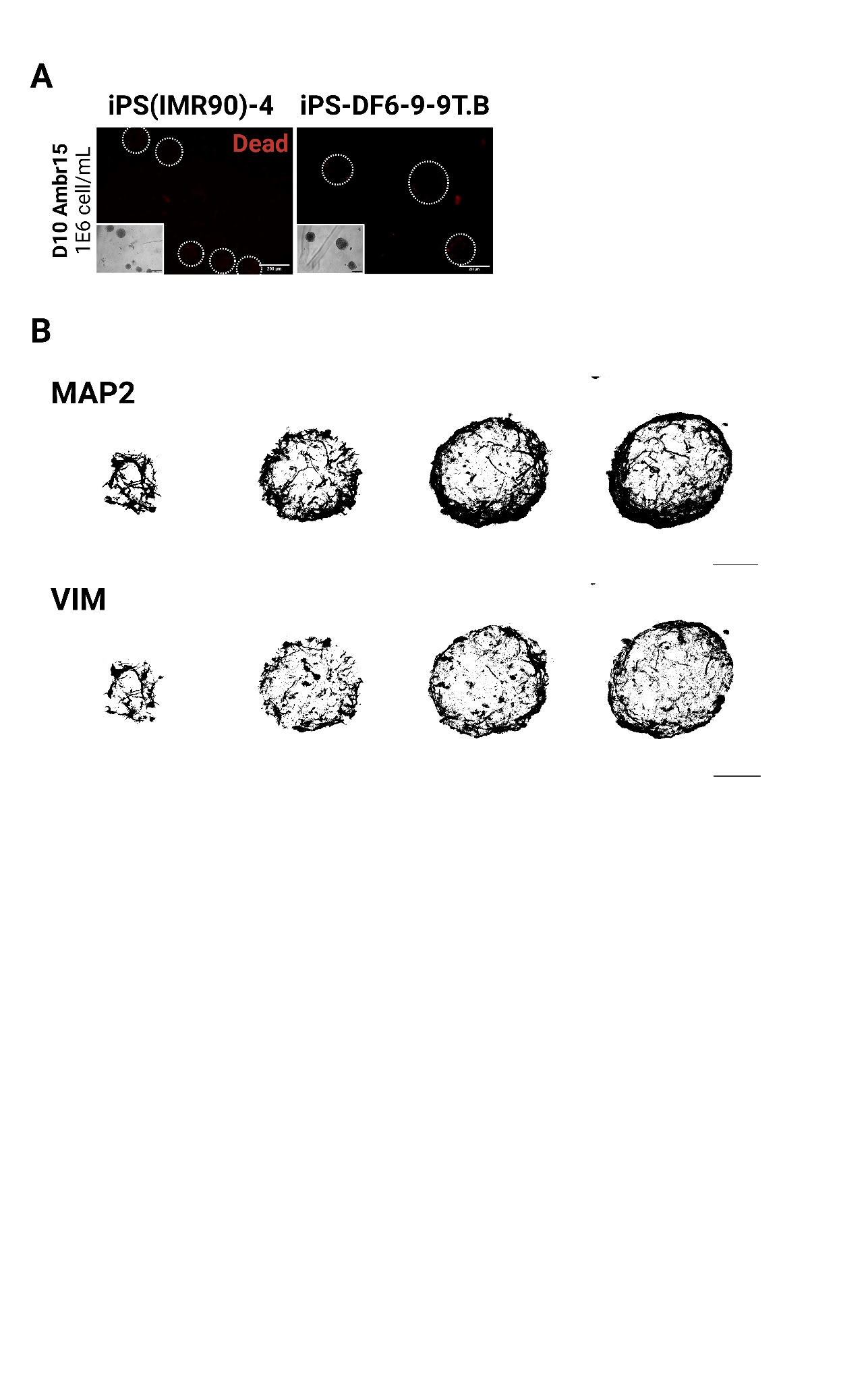


**Supplementary Figure 2 – Characterization of neurons and astrocytes within the iNSpheroids cultivated in the Ambr® 15 culture system, in different optical depths.**

**(A)** iNSpheroids derived from two hiPSC lines, iPS(IMR90)-4 and iPS-DF6-9-9T.B, cultured for 10-days in the Ambr® 15 culture system (Ambr15); cell dead was evaluated by incorporation of propidium iodine (PI, red). INSpheroid perimeter is identified as dashed circles (white), detected by the phase contrast microscopy (inset). Scale bar, 200 μm. **(B)** Immunofluorescence detection of MAP2 (upper panel, microtubule-associated protein 2, marker of the somatodendritic compartment of mature neurons) and vimentin (lower panel, intermediate filament of astrocytes), in iNSpheroids from the 0.5 condition, after 15 days of culture. Representation of the optical slices 6, 16, 26 and 36 with binary threshold masks (of the 36 that compose the z-stack depicted as maximum intensity projection in Figure 1B).

**Supplementary Figure 3
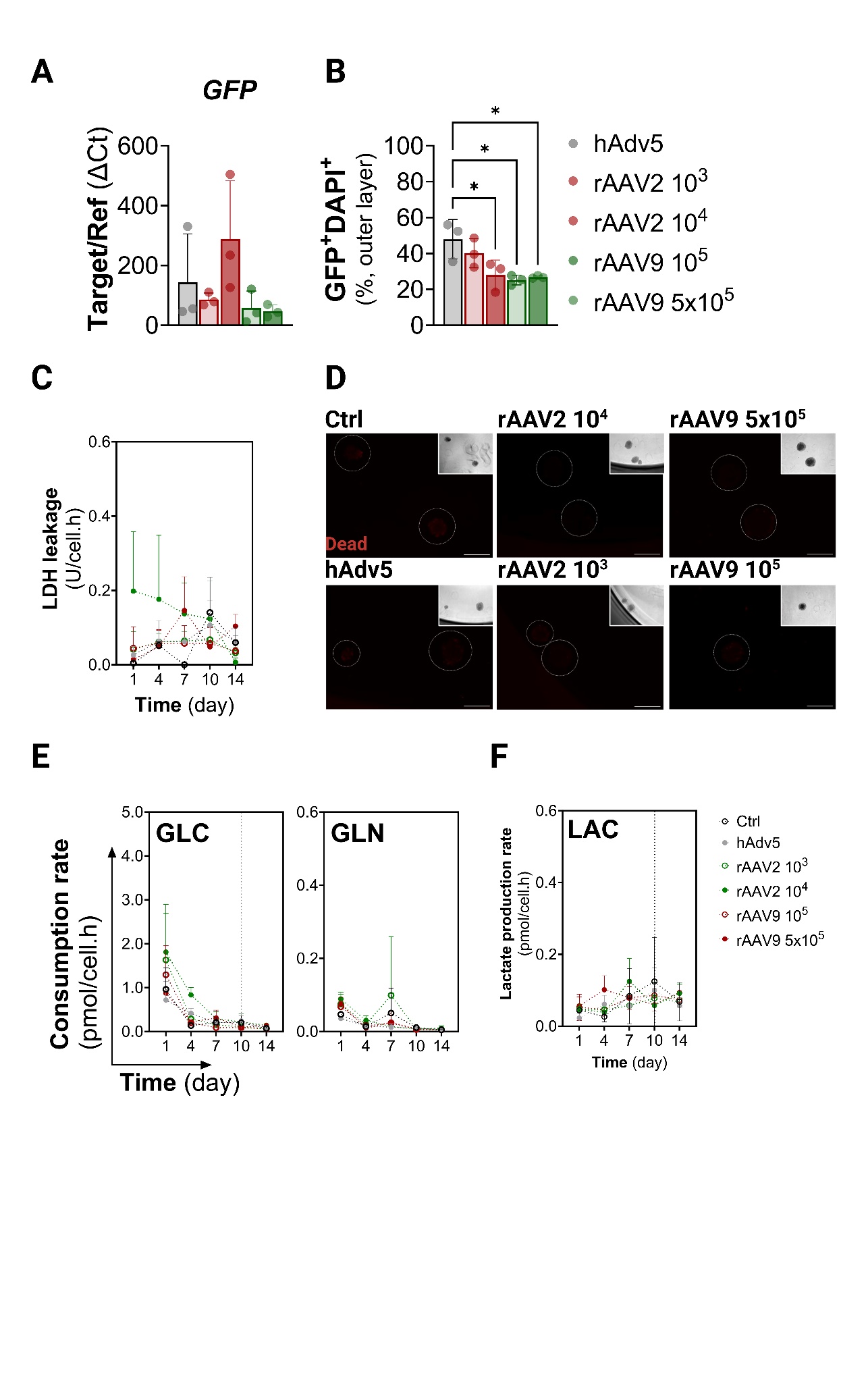
 - rAAV transduction of 3D hiPSC-derived iNSpheroids in the Ambr® 15 culture system**

**(A)** eGFP gene expression after 14 days of transduction. mRNA quantification in hAdv5 and rAAV transduced conditions were analyzed by RT-PCR. Data are shown as the ratio target, reference (ΔCt), considering housekeeping genes *GAPDH* and *RPL22*. Data are represented as mean ± SD, of three independent experiments. **(B)** Immunofluorescence-based quantification of transduction efficiency, for each viral vector and MOI: percentage of eGFP-labeled cells, of the outer layer cells in the iNSpheroid (identified by nuclei staining, DAPI). Data are represented as mean ± SD, of three independent experiments. For comparing three or more groups two-way ANOVA test with Šídák's correction was used **(A)**: ns, non-significant; * p<0.05; ** p<0.01, *** p<0.001. Viability assessment of the iNSpheroids transduced with the different viral vectors and MOIs, in comparison to the non-transduced control. The viability was assessed by **(C)** the specific LDH leakage to the culture supernatant, and **(D)** the incorporation of propidium iodine (PI, red) by dead cells. INSpheroid perimeter is identified as dashed circles, detected by the phase contrast microscopy. Scale bar, 100 μm. Specific **(E)** GLC and GLN consumption; and **(F)** LAC secretion. The vertical dashed line represents the time point of the glucose feeding. Data represented as mean ± SD of three independent experiments.


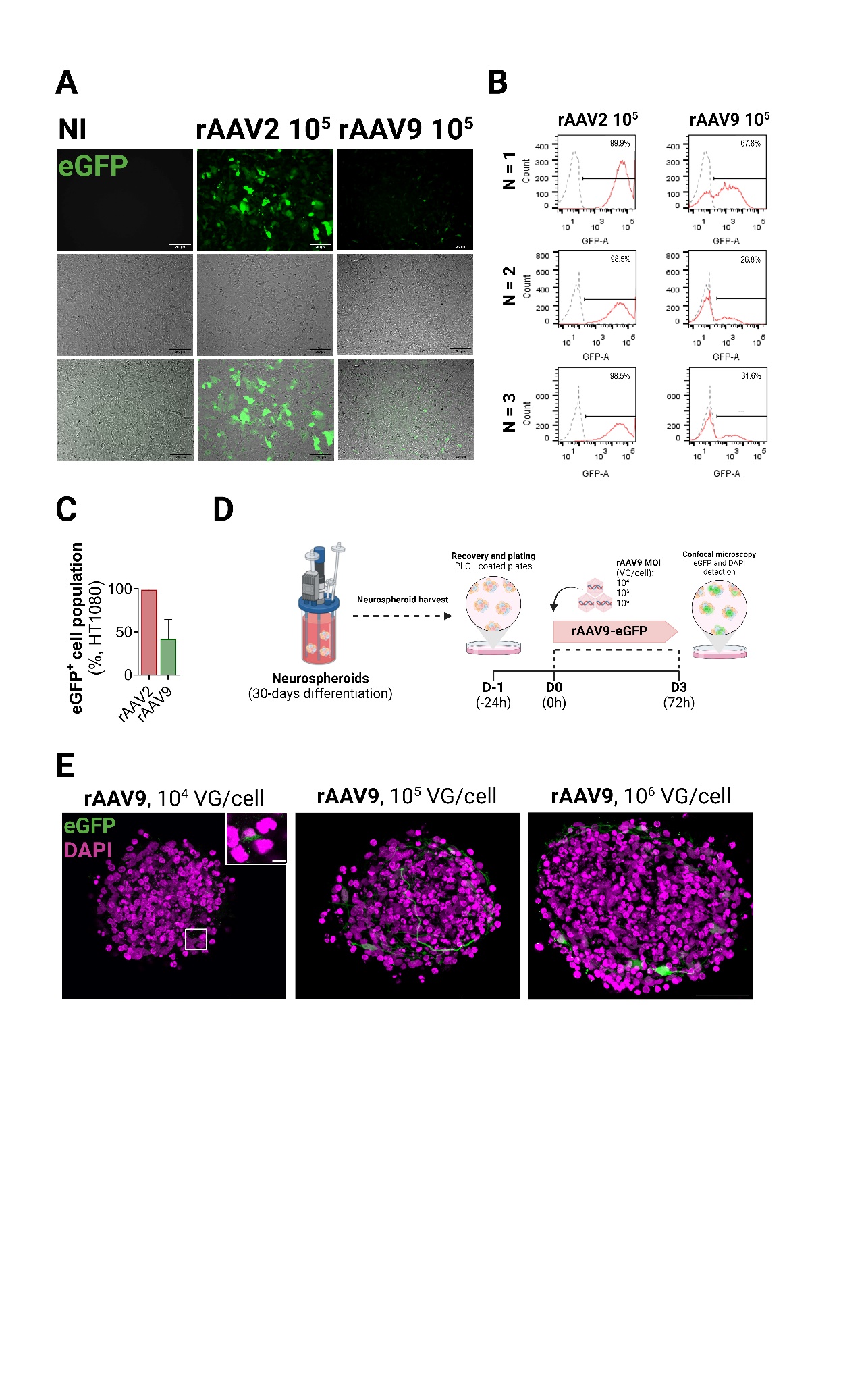


**Supplementary Figure 4 - rAAV transduction efficiency in static systems, namely 2D HT1080 and 3D iNSpheroids plated in PLOL substrate.**

Immunofluorescence- and flow cytometry-based qualitative and quantitative assessment of rAAV transduction capacity, in 2D cultured HT1080 cell line (rAAV9 and rAAV2) and for iNSpheroids plated in PLOL coating after STB control harvest. Immunofluorescence **(A)** and flow cytometry **(B, C)** detection of eGFP (green, green fluorescence protein) in 2D cultured HT1080 cell line, after 72 hours post-transduction with rAAV2 and rAAV9 at 10^5^ VG/cell, in comparison to non-infected (NI) control. Scale bar, 200 μm. **(B)** Flow cytometry histogram plots and **(C)** bar graph representation of the 3 independent experiments. **(D)** Schematic representation of the iNSpheroid harvest from the STB control to a PLOL-coated plate. Briefly, iNSpheroids are transferred and allowed to adhere for 24 hours, after which rAAV9 was inoculated at three different MOIs, 10^4^, 10^5^ and 10^6^. **(E)** Immunofluorescence detection of eGFP (green) and counterstained with DAPI (magenta). Scale bar, 50 and 5 μm (inset), maximum projection of 40 optical slices with 0.5 μm slice thickness.

# Supplemental information

## HT1080 cell culture

HT1080 cells (ATCC® CCL121) cells were cultured in high glucose (4.5 g/L) DMEM (Gibco Life Technologies) supplemented with 10% (v/v) fetal bovine serum (FBS, Gibco Life Technologies). Cells were incubated at 37 °C in a humidified atmosphere with 5% CO2.

## rAAV vector transduction and flow cytometry

HT1080 cells at 60–70% confluency was transduced with rAAV2 and rAAV9 vectors at a multiplicity of infection (MOI) of 1E5 VG/cell in 2% serum containing media. After 8 h incubation, medium was replaced by complete media. After 72 hours of transduction. HT1080 cells were imaged by fluorescence microscopy (DMI6000 B, Leica) and collected for flow cytometry (BD FACSCelesta™ Cell Analyzer, BD Biosciences) for eGFP expression assessment.

## Immunofluorescence microscopy: iNSpheroids plated in coverslips

Neurospheroids were harvested from the STB control, plated on PLOL-coated glass coverslips, and allowed to adhere for 6-8 hours. Afterwards, the samples were fixed in 4% paraformaldehyde (PFA) + 4% sucrose in PBS for 20 minutes at room temperature (RT) and washed three times with PBS. Immunostaining protocol was performed as described in the methods section, in the manuscript. Coverslips were mounted in ProLong™ Gold Antifade Mountant (Life Technologies). Images were acquired on a Zeiss LSM 880-point scanning confocal microscope controlled with the Zeiss Zen 2.3 (black edition) software. The obtained images were processed using ImageJ software and only linear manipulations were performed (Schindelin et al., 2012).
